# The blooming of long-read sequencing reforms biomedical research

**DOI:** 10.1186/s13059-022-02604-2

**Published:** 2022-01-12

**Authors:** Kin Fai Au

**Affiliations:** 1 Department of Biomedical Informatics, The Ohio State University, Columbus, OH 43210 USA; 2 Biomedical Informatics Shared Resources, The Ohio State University, Columbus, OH 43210 USA

Compared to Next Generation Sequencing (NGS), PacBio and nanopore sequencing offer ultra-long reads for single DNA/RNA molecules. These long reads are very informative to address omics problems where large-range complexity is involved, such as genome assembly, haplotyping, complex variant calling, and gene isoform identification. The single-molecule feature of long-read sequencing also allows for simultaneous measurements of base modifications together with other omics features, such as genomics and transcriptomics. This gives us unprecedented views on biomedical problems that have, until now, remained poorly characterized [1, 2]. Moreover, the accuracy, accessibility, and cost efficiency of long-read sequencing are improving dramatically, which boosts the long read-based research in many topics.

Therefore, we are not only in the midst of a new revolution in sequencing technology but also the next revolution in biomedical research. To timely and fully utilize the unique benefits of this technological breakthrough, there is much enthusiasm to develop new experimental and computational methods and apply long-read sequencing to diverse biomedical contexts. This special issue collects the latest work of several typical types of long read-based research in genomics, transcriptomics, and cancer diagnosis. Some of them aim to improve the existing analyses that rely on the NGS-based methods, and some others are unique applications for long-read sequencing, such as nanopore adaptive sampling.

Genome assembly is one of the earliest and the most popular applications of long reads. As they cover many single nucleotide polymorphisms (SNPs), long reads are useful to advance genome assembly to the haplotype resolution. The new software phasebook adapts a divide-and-conquer strategy to improve the coverage of haplotype-resolved de novo genome assembly [3]. Since sample preparation, such as high-molecular-weight DNA extraction, could influence the data quality and thus assembly significantly, the end-to-end plant genome assembly workflow LeafGO optimizes the steps from sample preparation to computational analysis [4]. In addition to the application for the diploid genomes, LeafGO was also tested in the allotetraploid genome of *Arachis hypogaea*. Improved haplotype-resolved assembly is very beneficial to many research and applications, such as precision medicine and evolutionary biology. For example, Xue et al. used PacBio HiFi reads to create a high-quality and nearly gap-free diploid genome of zig-zag eel so that they could perform a high-resolution comparison of the homomorphic pair of the sex chromosomes to investigate their recombination and differentiation [5]. The power of long reads was also shown in characterizing structural variations and repetitive elements [6, 7].

In the field of transcriptomics, abundance estimation is the basis of many other analyses, so Hu et al. published LIQA, a more sophisticated method for gene isoform quantification by long reads other than simply using read counts as the expression index in the previous studies [8]. In parallel, the interests of single-cell sequencing are emerging in the field of long read-based transcriptomics research. In particular, Tian et al. optimized a droplet-based protocol for generating high-quality single-cell sequencing data of both short reads and long reads and also established a bioinformatics pipeline FLAMES for comprehensive analyses, such as isoform identification and mutation detection in single cells [9]. Considering cost efficiency, Rebboah et al. reported a protocol LR-Split-seq that integrates the combinatorial barcoding of Split-seq with long-read sequencing to achieve differential gene isoform expression analysis at the single-cell level [10]. The application of LR-Split-seq to the C2C12 myogenic system found the distinct patterns of alternative transcription start sites and/or alternative internal exon usage in different cell clusters. Besides cDNA sequencing, nanopore sequencing can measure native RNA molecules directly, so Schulz et al. were able to revisit the reliability of exitron identification by comparing the data of direct RNA sequencing and cDNA sequencing [11]. They found that dozens of exitrons may be artifacts of reverse transcription, highlighting the value and importance of validation by direct RNA sequencing.

To leverage the time-/cost-efficiency of nanopore sequencing for clinical usage, Thirunavukarasu et al. developed a cancer screening protocol “Oncogene Concatenated Enriched Amplicon Nanopore Sequencing (OCEANS)” targeting the somatic mutations with low variant allele frequency [12]. They demonstrated the accuracy by applying the specific panels of recurrent mutations to four cancer types and showed it a possible measure for rapid and affordable clinical sequencing.

A few new efforts are specific for long-read sequencing. For example, adaptive sampling is a unique application of nanopore sequencing to enrich target elements of interest—real-time analysis of the raw electrical signals determines whether the molecules are ejected, or the data collection continues. Bao et al. developed the first deep learning-based software SquiggleNet to improve the analysis speed and computing memory usage [13]. Martin et al. established a mathematical model to evaluate how a set of factors, such as molecule length, influence the enrichment performance, so that the output can be predicted and a guideline of adaptive sampling was also provided [14].

Considering the rapid growth of computational methods, experimental techniques, and applications, benchmarking is a critical type of effort to optimize and promote the usage of the long-read sequencing, especially for many starters with limited experience. For instance, Chen et al. developed a computational platform Inspector to evaluate genome assembly [15]. Because of the large variance of long-read sequencing data quality, such as read length and error profile, the performance of assemblers was examined in different data scenarios (e.g., PacBio CLR and HiFi reads and nanopore data). It is indeed a good practice to benchmark long read-based methods, and doing so can provide more specific guidelines for data collection and software selection. Liu et al. completed a comprehensive survey for nanopore sequencing-based 5mC detection across different genomic contexts, CpG site coverage, and computational resources [16]. In addition to the single-site resolution, the “per-read” accuracy, i.e., detection at the single-molecule level, was also tested, which is a new view for advancing epigenetics research. Like the era of NGS, consortium-scale efforts of method benchmarking and construction of omics landscapes will be very beneficial to the community of long-read sequencing by providing useful analysis guidelines and valuable data resources. For instance, the Long-read RNA-seq Genome Annotation Assessment Project (LRGASP) Consortium is now organizing a large-scale survey of different protocols and software for long read-based RNA-seq [17].

Although it is not possible to include all significant research of long-read sequencing within a single special issue, this collection of articles represents the emerging interests and trends of long read-based method development and applications. We foresee much more creative and impactful research of long-read sequencing in the coming years.
